# Supplementary material for: Butyrate in Human Milk: Associations with Milk Microbiota, Milk Intake Volume, and Infant Growth
Source: Nutrients. 2023 Feb 11;15(4):916. doi: 10.3390/nu15040916 (PMC9963357; doi:10.3390/nu15040916)
Supplement: Supplementary file 1 [file nutrients-15-00916-s001.zip › nutrients-2127514-supplementary.pdf]

Supplementary materials

Supplementary Table S1 Study design

Biological sampling/measurement	Age						
	Birth	2 weeks	4-6 weeks	6 weeks	3 months	6 months	12 months
Recruitment	v						
BM intake volume			v				
Liquid BM sampling for butyrate analysis	v	v		v	v	v	v
Sterile sampling of BM for microbiome analysis				v			
Infant anthropometric measurement	v	v		v	v	v	v

Supplementary Table S2 Number of subjects with sample availability for each experiment

Experiment	Number of subjects
Total included in longitudinal analyses between HM butyrate and growth	71
• HM butyrate measured at 6 weeks	59
• HM butyrate intake estimated between 4-6 weeks	47
Total included in microbiome study	69
• With HM butyrate concentrations	56

Supplementary Table S3 The associations between maternal/infant factors and HM butyrate concentration at 6 weeks

**Factors**	**HM butyrate concentration at 6 weeks**	
	**Correlation coefficient***	**p**
Maternal age at delivery (years)	0.08	*0.55*
Maternal pre-pregnancy BMI (kg/m^2)	-0.02	*0.87*
Maternal height (cm)	-0.24	*0.08*
Parity (primiparous vs multiparous)	-0.18	*0.28*
Maternal ethnicity (White European vs others)	-0.23	*0.33*
Gestational age (weeks)	0.07	*0.59*
Infant sex (male vs female)	-0.01	*0.93*
Exclusive breastfeeding duration ($\geq$12 weeks vs <12 weeks)	-0.36	*0.06*

*Pearson R for continuous and point-biserial correlation for categorical variables
BMI, body mass index; IMD, index of multiple deprivation

Supplementary Table S4 Cross-sectional associations between HM butyrate concentrations and infant growth parameters

**Growth parameters**	**Butyrate concentration (mg/100 mL)**	
	**B+SE**	***p***
**2 weeks**		
Weight SDS	-0.18+0.15	*0.25*
Length SDS	-0.06+0.25	*0.81*
BMI SDS	-0.22+0.24	*0.36*
Mean skinfolds SDS	-0.11+0.29	*0.7*
**6 weeks**		
Weight SDS	-0.4+0.19	***0.04***
Length SDS	-0.22+0.21	*0.29*
BMI SDS	-0.4+0.23	*0.09*
Mean skinfolds SDS	-0.44+0.24	*0.08*
**3 months**		
Weight SDS	-0.21+0.18	*0.23*
Length SDS	-0.18+0.12	*0.17*
BMI SDS	-0.15+0.2	*0.47*
Mean skinfolds SDS	0.03+0.18	*0.85*
**6 months**		
Weight SDS	0.05+0.21	*0.81*
Length SDS	-0.06+0.15	*0.7*
BMI SDS	0.12+0.23	*0.63*
Mean skinfolds SDS	0.05+0.21	*0.81*

Values are unstandardized regression coefficients (B) and the corresponding standard errors (SE)
All subjects were exclusively breastfed in the first 6 weeks of life
Models are adjusted for infant sex, gestational age, postnatal age at visit, and birth weight SDS. Models at 3- and 6- months are additionally adjusted feeding history (EBF for 6-11 weeks vs EBF for ≥12 weeks)
Associations with *p<0.05* are highlighted in bold

Supplementary Figure S1 HM butyrate concentration based on EBF duration
0=EBF duration 6 weeks-3 months, 1=EBF duration ≥3 months

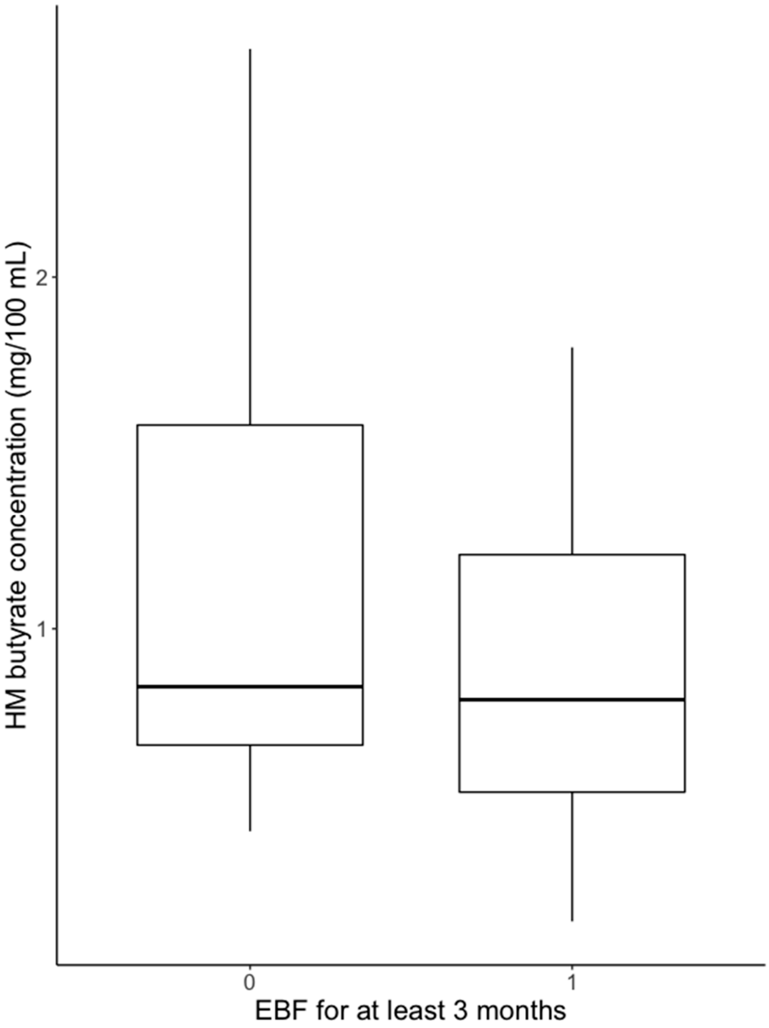

HM=human milk
EBF=exclusive breastfeeding

Supplementary Figure S2 Average composition of HM microbiota

The fraction of 16S rRNA reads (in %) attributed to specific taxonomic level is given below the taxon name. Figure is generated using software described in Sundquist A, Bigdeli S, Jalili R, Druzin ML, Waller S, Pullen KM, El-Sayed YY, Taslimi MM, Batzoglou S, Ronaghi M. Bacterial flora-typing with targeted, chip-based Pyrosequencing. BMC Microbiol. 2007 Nov 30;7:108. doi: 10.1186/1471-2180-7-108. PMID: 18047683; PMCID: PMC2244631.

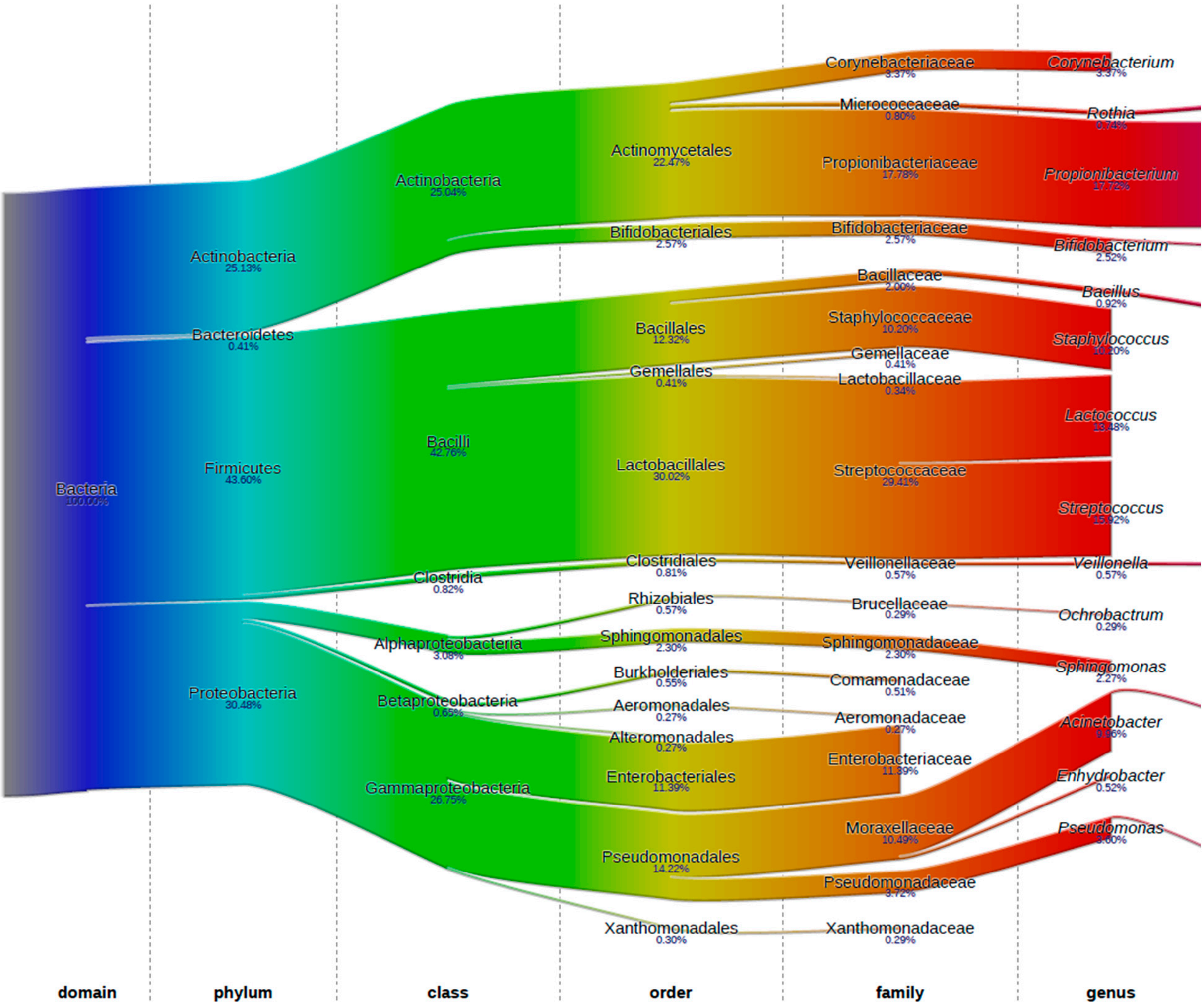

Supplementary Figure S3 Longitudinal weight gain trajectories based on HM butyrate concentration

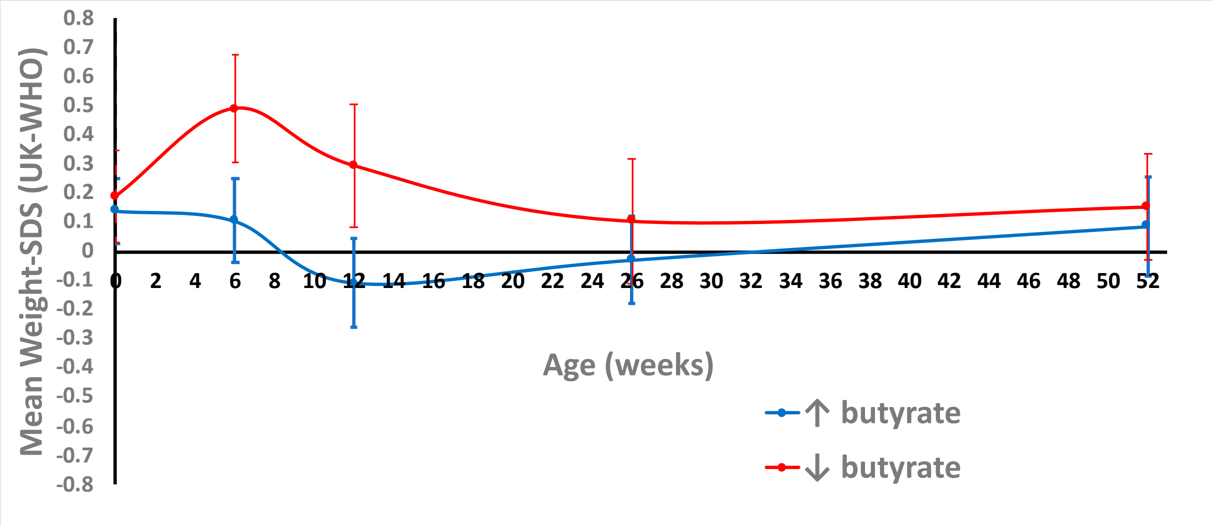

Following UK guidelines, weight values were converted to sex and age-adjusted SDS using the British 1990 growth reference at birth and subsequently using WHO International Growth Standard.
Grey shaded-square represents minimum EBF period for all participants (all subjects received EBF for at least 6 weeks).
EBF=exclusive breastfeeding, SDS=standard deviation scores
